# Multiple-Ascending-Dose Phase 1 Clinical Study of the Safety, Tolerability, and Pharmacokinetics of CRS3123, a Narrow-Spectrum Agent with Minimal Disruption of Normal Gut Microbiota

**DOI:** 10.1128/AAC.01395-19

**Published:** 2019-12-20

**Authors:** Barbara K. Lomeli, Hal Galbraith, Jared Schettler, George A. Saviolakis, Wael El-Amin, Blaire Osborn, Jacques Ravel, Keith Hazleton, Catherine A. Lozupone, Ronald J. Evans, Stacie J. Bell, Urs A. Ochsner, Thale C. Jarvis, Shahida Baqar, Nebojsa Janjic

**Affiliations:** a Quintiles Phase One Services, Overland Park, Kansas, USA; b DynPort Vaccine Company LLC, Frederick, Maryland, USA; c Division of Microbiology and Infectious Diseases, National Institute of Allergy and Infectious Diseases, National Institutes of Health, Bethesda, Maryland, USA; d Department of Microbiology and Immunology, University of Maryland School of Medicine, Baltimore, Maryland, USA; e Institute for Genome Sciences, University of Maryland School of Medicine, Baltimore, Maryland, USA; f Division of Pediatric Gastroenterology, Hepatology and Nutrition, University of Colorado Anschutz Medical Campus, Aurora, Colorado, USA; g Division of Biomedical Informatics and Personalized Medicine, University of Colorado Anschutz Medical Campus, Aurora, Colorado, USA; h Crestone, Inc., Boulder, Colorado, USA

**Keywords:** CRS3123, *Clostridioides difficile*, MAD study, antibiotic, gut microbiota, microbiome, narrow spectrum, phase 1, sporulation, toxin production

## Abstract

CRS3123 is a novel small molecule that potently inhibits methionyl-tRNA synthetase of Clostridioides difficile, inhibiting C. difficile toxin production and spore formation. CRS3123 has been evaluated in a multiple-ascending-dose placebo-controlled phase 1 trial. Thirty healthy subjects, ages 18 to 45 years, were randomized into three cohorts of 10 subjects each, receiving either 200, 400, or 600 mg of CRS3123 (8 subjects per cohort) or placebo (2 subjects per cohort) by oral administration twice daily for 10 days. CRS3123 was generally safe and well tolerated, with no serious adverse events (SAEs) or severe treatment-emergent adverse events (TEAEs) reported. All subjects completed their assigned treatment and follow-up visits, and there were no trends in systemic, vital sign, or laboratory TEAEs. There were no QTcF interval changes or any clinically significant changes in other electrocardiogram (ECG) intervals or morphology. CRS3123 showed limited but detectable systemic uptake; although absorption increased with increasing dose, the increase was less than dose proportional. Importantly, the bulk of the oral dose was not absorbed, and fecal concentrations were substantially above the MIC_90_ value of 1 μg/ml at all dosages tested. Subjects receiving either of the two lower doses of CRS3123 exhibited minimal disruption of normal gut microbiota after 10 days of twice-daily dosing. CRS3123 was inactive against important commensal anaerobes, including *Bacteroides*, bifidobacteria, and commensal clostridia. Microbiome data showed favorable differentiation compared to other CDI therapeutics. These results support further development of CRS3123 as an oral agent for the treatment of CDI. (This study has been registered at Clinicaltrials.gov under identifier NCT02106338.)

## Abstract (precis)

CRS3123 is a novel small molecule that potently inhibits methionyl-tRNA synthetase of Clostridioides difficile, inhibiting C. difficile toxin production and spore formation. CRS3123 has been evaluated in a multiple-ascending-dose placebo-controlled phase 1 trial.

## INTRODUCTION

Clostridioides difficile infection (CDI) is currently classified as an urgent threat by the CDC as the leading cause of antibiotic-associated diarrhea among hospitalized patients (1, 2) and the most common cause of health care-associated infections in the United States (3, 4). CDI is caused by Gram-positive, spore-forming, and toxin-producing strains of C. difficile bacteria, which are ubiquitous in the environment. The disruption of colonic epithelium is mediated by toxins A and B (TcdA and TcdB), with TcdB being the main virulence factor in CDI (5–7). Predisposition to CDI is caused by disruption of healthy intestinal microbiota, typically following exposure to broad-spectrum antibiotics (8), chemotherapeutic agents, and/or radiation treatment (9), leading to decreased colonization resistance. Infection with hypervirulent and drug-resistant strains of C. difficile, such as ribotypes 027 and 078, is generally associated with more severe disease (10, 11).

Treatment options for CDI remain limited. Metronidazole and vancomycin, which currently account for the vast majority of prescriptions for CDI, are widely regarded as suboptimal therapies due to their broad spectra and the associated high (20 to 40%) recurrence rates (12–15). The incidence of primary CDI has risen substantially over the last 2 decades, but the increase in recurrence rates has emerged as an even more alarming issue; recurrence necessitates retreatment which is often associated with increasingly poor prognosis, driven by an increasing probability of recurrence with each episode (16–19).

Until recently, vancomycin was the only FDA-approved antibiotic for the treatment of CDI. Its widespread use has led to growing concerns about selection for vancomycin resistance, especially among enterococci (vancomycin-resistant enterococci [VRE]) and staphylococci (vancomycin-resistant Staphylococcus epidermidis [VRSE], vancomycin-intermediate Staphylococcus aureus [VISA], and vancomycin-resistant S. aureus [VRSA]). To protect vancomycin from overuse (20), metronidazole, which has never been approved for the treatment of CDI, has been used as an off-label alternative to vancomycin for mild to moderate CDI (21, 22). Now clearly established as an inferior treatment option, in terms of both primary cure and recurrence rates (1, 23, 24), metronidazole is no longer recommended in the latest Infectious Diseases Society of America (IDSA) and Society for Healthcare Epidemiology of America (SHEA) guidelines as the initial therapy for nonsevere or severe CDI in adults, except in special cases (1). The latest drug to receive approval for CDI, fidaxomicin, has a somewhat narrower spectrum and consequently lower overall recurrence rate than those of vancomycin, but only for infections caused by nonepidemic strains (25, 26).

Potential alternatives to antibiotic treatment of CDI have received considerable attention recently, such as the use of fecal microbiota transplant (FMT) and TcdB-binding monoclonal antibody bezlotoxumab (7, 27, 28). Although such treatments have shown efficacy as adjuncts to antibiotic therapy in reducing the incidence of recurrence, antibiotics remain the primary treatment option for the initial episode of CDI, as well as for the first recurrences (1). Therefore, the need for narrow-spectrum antibacterial agents that have the potential to further reduce recurrence remains high.

C. difficile bacteria in stationary phase remain metabolically active and continue to produce toxins. As a class, protein synthesis inhibitors may have an advantage over other antibiotic classes that interfere with metabolic pathways less active in stationary-phase bacteria, such as inhibitors of DNA replication, RNA synthesis, or cell wall synthesis.

CRS3123 is a fully synthetic small-molecule antibacterial drug candidate with a novel mechanism of action that potently inhibits type 1 methionyl-tRNA synthetase (MetRS), an essential component of protein translation (29, 30). CRS3123 is active only in bacteria that express type 1 MetRS, which include C. difficile, Clostridium perfringens, and many aerobic Gram-positive bacteria, such as staphylococci, enterococci, and streptococci. Bacterial strains that express type 2 MetRS show virtually no susceptibility to CRS3123 and include most Gram-negative bacteria and many constituents of the normal intestinal microbiota such as *Bacteroides*, bifidobacteria, actinobacteria, and lactobacilli (31). As a protein synthesis inhibitor, CRS3123 inhibits C. difficile toxin production and sporulation, and it is superior to vancomycin in protecting hamsters against recurrence in an *in vivo* model of CDI (29, 30). In addition, CRS3123 has exhibited low systemic absorption following oral dosing in preclinical testing, as well as in a first-in-human single-ascending-dose (SAD) study at doses ranging from 100 mg to 1,200 mg, in which the drug was also safe and well tolerated (32). Here we report the results of a randomized, double-blind, placebo-controlled, multiple-ascending-dose (MAD) phase 1 clinical trial in healthy subjects who received 200 mg, 400 mg, or 600 mg twice-daily (BID) oral doses of CRS3123 or placebo for 10 days, the intended dose frequency and duration of therapy in CDI patients. We show that CRS3123 was generally safe and well tolerated at all doses tested, with limited but detectable systemic uptake and high fecal concentrations. We also show limited impact of CRS3123 on the intestinal microbiota of healthy subjects participating in this study based on the evaluation of fecal microbiome using 16S rRNA gene sequencing. The studies conducted to date warrant the evaluation of CRS3123 in the patient population as a promising treatment for primary and recurrent CDI.

## RESULTS

Here we report the results of a multiple-ascending-dose, single-center, randomized, placebo-controlled, double-blind study phase 1 study of CRS3123.

### Demographics.

**FIG 1:**
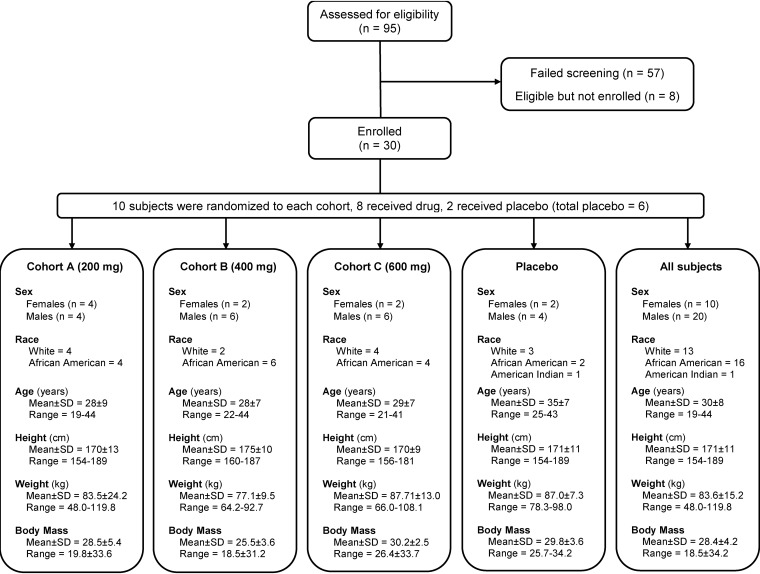
Study design and demographics.

Thirty healthy male and female subjects, ages 18 to 45 years, were randomized into three ascending-dose cohorts: 200, 400, and 600 mg twice daily for 10 days (cohorts A, B, and C) (Fig. 1). No subjects reported clinically significant medical history findings. Overall, more than 60% of the enrolled subjects were males (66.7%), and the overall mean age of the subjects was 30 years (Fig. 1; see also Table S1 in the supplemental material).

### Pharmacokinetics.

**FIG 2:**
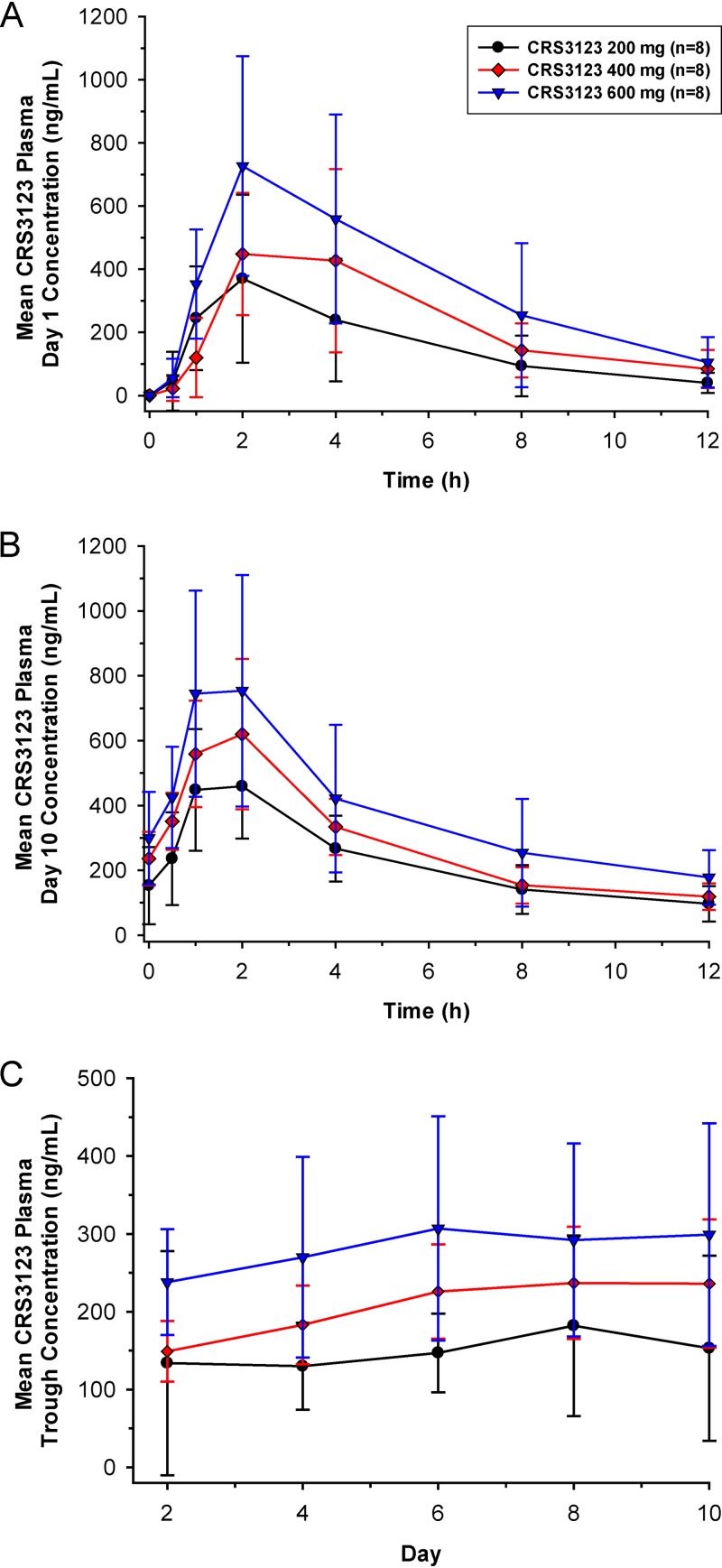
Mean (±SD) plasma CRS3123 concentration-time profiles on day 1 following dose 1 (A) and day 10 following dose 19 (B) and mean (±SD) plasma CRS3123 trough concentrations for the three cohorts (C). Trough concentrations samples were acquired immediately prior to doses 3 (day 2), 7 (day 4), 11 (day 6), 15 (day 8), and 19 (day 10).

**FIG 3:**
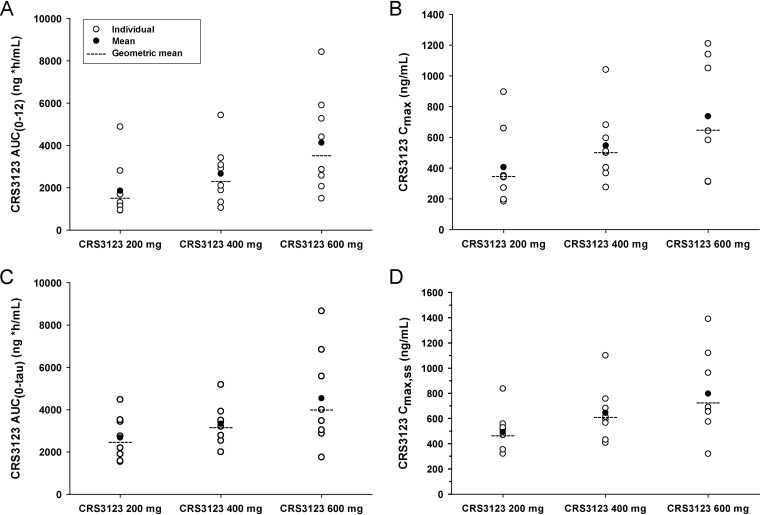
Individual, mean, and geometric mean plasma CRS3123 AUC_0–12_ on day 1 (A) and day 10 (C) and *C*_max_ on day 1 (B) and day 10 (D) for the three cohorts.

**(i) Plasma.** After the first dose of CRS3123 on day 1, median time to maximum concentration (*T*_max_) ranged from 2 to 3 h and geometric mean half-life (*t*_1/2_) ranged from 3 to 4 h across the 3 dose groups (Fig. 2A). Geometric mean CRS3123 area under the concentration-time curve from 0 to 12 h (AUC_0–12_) and maximum concentration (*C*_max_) following first dose administration increased approximately 2.3-fold and 1.9-fold, respectively, with a 3-fold increase in dose from 200 mg to 600 mg (Fig. 3A and B). Plasma CRS3123 pharmacokinetic (PK) parameters on day 1 following a single dose are summarized by treatment in Table 1 and Table S2.

**TABLE 1:** Summary of CRS3123 PK parameters in plasma, stool, and urine

Pharmacokinetic parameter	200 mg		400 mg		600 mg	
	Day 1	Day 10	Day 1	Day 10	Day 1	Day 10
Plasma						
AUC (ng·h/ml)^*a*^^,^^*b*^	1,550 (64.1)	2,500 (41.3)	2,340 (57.6)	3,200 (29.4)	3,560 (64.1)	4,030 (55.6)
*C*_max_ (ng/ml)^*a*^^,^^*c*^	352 (59.4)	470 (32.3)	507 (42.5)	615 (32.4)	654 (57.9)	731 (47.9)
*T*_max_ (h)^*d*^^,^^*e*^	2.0 (0.5, 4.0)	1.5 (1.0, 2.0)	3.0 (2.0, 4.0)	2.0 (1.0, 2.0)	2.0 (1.0, 4.0)	1.0 (1.0, 2.0)
*t*_1/2_ (h)^*d*^^,^^*e*^	3.0 (2.7, 3.7)	4.6 (4.1, 7.0)	3.6 (2.9, 4.3)	5.0 (3.4, 7.7)	2.9 (2.6, 4.0)	6.1 (5.4, 7.3)
Stool						
*C*_stool_ (μg/g)^*d*^^,^^*f*^	NC^*i*^	2,115 (966, 4,430)	NC	5,390 (2,920, 8,700)	NC	8,280 (2,200, 14,000)
*f*_e_,_stool_^*d*^^,^^*h*^	NC	24.9 (1.9, 68.8)	NC	52.0 (3.6, 87.6)	NC	43.1 (11.0, 125.8)
Urine						
*A*_e_,_urine_ (μg)^*d*^^,^^*g*^	2,300 (1,550, 3,060)	3,380 (2,640, 4,920)	4,170 (2,380, 8,220)	5,260 (4,110, 7,260)	4,250 (2,860, 8,900)	6,630 (3,320, 9,880)
*f*_e_,_urine_^*d*^^,^^*h*^	1.2 (0.78, 1.5)	1.7 (1.3, 2.5)	1.1 (0.60, 2.1)	1.3 (1.0, 1.8)	0.71 (0.48, 1.5)	1.1 (0.55, 1.7)

a Geometric mean (percent coefficient of variation [CV]).

b AUC_0–12_ for day 1, AUC_0–tau_ for day 10.

c *C*_max_ for day 1, *C*_max,ss_ for day 10.

d Median (minimum, maximum).

e *T*_max_ for day 1, *T*_max,ss_ for day 10.

f *C*_stool_ for day 1, *C*_stool,ss_ for day 10.

g Amount excreted (*A*_e_): *A*_e,0–12_ for day 1, *A*_e,0–12,ss_ for day 10.

h Fraction excreted (*f*_e_): *A*_e,0–12_ for day 1, *A*_e,0–12,ss_ for day 10.

i NC, not calculated.

Following 10 days of twice-daily administration, median *T*_max_ at steady state (*T*_max,ss_) ranged from 1 to 2 h and geometric mean *t*_1/2,ss_ ranged from 5 to 6 h across the three CRS3123 dose groups (Fig. 2B). Across the dose range studied, geometric mean accumulation ratio for AUC (RAUC) ranged from 1.1 to 1.6 and geometric mean accumulation ratio for *C*_max_ (R*C*_max_) ranged from 1.1 to 1.3 (Table S3). Based on the mean plasma trough concentration-time profiles (Fig. 2C), there was minimal accumulation of CRS3123 following multiple twice-daily dosing for 10 days. This finding is consistent with the relatively short t_1/2,ss_ in relationship to the 12 h dosing interval. Plasma CRS3123 PK parameters on day 10 following multiple dosing are summarized in Table 1 and Table S3.

The increase in plasma exposure was less than proportional to the increase in dose across the dose range studied, at both day 1 and day 10. Dose proportionality of CRS3123 PK parameters following a single dose (AUC_0–12_ and *C*_max_ on day 1) and multiple doses (AUC_0–tau_ and *C*_max,ss_ on day 10) over the administered dose range were quantified statistically using a power model ([AUC_0–12_ and *C*_max_ on day 1; AUC_0–tau_ and *C*_max_ on day 10] = α × dose^β^) for each PK parameter on log scale as the dependent variable and the logarithm of the dose as the independent variable. The slope (β) estimates were well below one, indicating a less than proportional increase in exposure.

**FIG 4:**
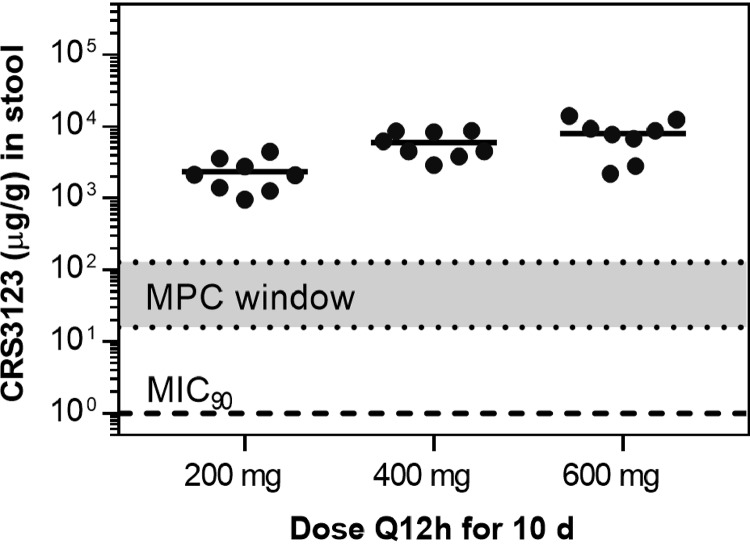
Accumulation of CRS3123 in the GI tract as measured in the stool.

**(ii) Fecal PK.** Following single-dose and multiple-dose administration of CRS3123, a substantial fraction of administered CRS3123 was retained in the gastrointestinal (GI) tract and excreted in feces (Table 1 and Table S4). Based on the fecal concentrations observed on day 10, mean *f*_e,feces,ss_ (see Materials and Methods) values under steady-state conditions were at least one-third, one-half, and two-thirds of the twice-daily doses of 200 mg, 400 mg, and 600 mg, respectively (Table 1, Table S4). Since this was not a mass balance study and the fecal sample size varied by over 2 orders of magnitude between subjects (ranging from 1.74 to 194.7 g during the 24-h collection period), these *f*_e,feces,ss_ values are approximate, yet consistent with fecal elimination being the major route of elimination for CRS3123. High levels in feces were observed at all three dose groups, with median values of 2,115 μg/g (range, 966 to 4,430), 5,390 μg/g (range, 2,920 to 8,700), and 8,280 μg/g (range, 2,200 to 14,000) following 200-, 400-, and 600-mg twice-daily doses (Fig. 4).

**(iii) Urine PK.** We have demonstrated previously that a small fraction of the orally administered dose of CRS3123 is metabolized into glucuronide conjugates detectable in urine (32). Therefore, we have converted the glucuronide conjugates into parent CRS3123 prior to urine analysis, so that the reported values reflect the sum of the free (parent) drug and its glucuronide metabolites. Renal elimination of CRS3123 as free drug and glucuronide conjugates was minimal and accounted for less than 2% of the administered dose. Following a single dose on day 1, approximately 1% of the dose was excreted in urine across the 3 dose groups as free drug and glucuronide conjugates. Following multiple dosing for 10 days, between 1% and 2% of the dose was excreted in urine across the 3 dose groups as free drug and glucuronide conjugates (Table 1 and Table S5).

### Safety assessment.

At all three doses tested, CRS3123 administered orally twice daily for 10 days was generally safe and well tolerated, with no deaths, other serious adverse events (SAEs), or treatment-emergent adverse events (TEAEs) reported. No subjects were withdrawn from the study due to TEAEs. There were no trends in systemic, vital sign, or laboratory TEAEs. The majority of TEAEs reported in the study were of mild severity. There was no prolongation of the QTcF interval or any clinically significant changes in other electrocardiogram (ECG) intervals or morphology. Treatment-related AEs were defined as TEAEs for which there was reasonable evidence to suggest a causal relationship between the study product and the AE. There were no treatment-related trends evident in the observed or change-from-baseline mean laboratory values and no clinically significant physical examination findings in this study.

Among the treatment-related AEs, the proportion of subjects receiving a placebo (33.3% subjects) reporting at least one treatment-related AE was higher than in the active groups (12.5% subjects). No subject in cohort A (200 mg of CRS3123) reported any treatment-related AEs. Two subjects in cohort B (400 mg CRS3123) reported at least one treatment-related AE: subject 2003 reported a mild treatment-related AE of diarrhea on day 2 which resolved the same day, and subject 2004 reported a mild treatment-related AE of dysgeusia on day 4 which resolved the following day. One subject (3004) in cohort C had a mild treatment-related AE of increased alanine aminotransferase (ALT) on day 6 which resolved 12 days later. Subject 1010 (cohort A, placebo) had a mild treatment-related AE of increased lipase on day 10, and subject 2006 (cohort B, placebo) reported a mild treatment-related AE of diarrhea on day 5; both of these treatment-related AEs resolved the following day. Summaries of all TEAEs and treatment-related AEs by system organ class and preferred term for each treatment (safety population) are presented in Tables 2 and 3, respectively.

**TABLE 2:** Summary of all treatment-emergent adverse events by system organ class and preferred term for each treatment (safety population)^*a*^

System organ class/preferred term	No. (%) of subjects				
	Placebo (*n* = 6)	Cohort A (200 mg Q12h) (*n* = 8)	Cohort B (400 mg Q12h) (*n* = 8)	Cohort C (600 mg Q12h) (*n* = 8)	All active (*n* = 24)
Subjects with TEAEs	6 (100.0)	8 (100.0)	6 (75.0)	6 (75.0)	20 (83.3)
Ear and labyrinth disorders	0 (0.0)	1 (12.5)	0 (0.0)	0 (0.0)	1 (4.2)
Ear discomfort	0 (0.0)	1 (12.5)	0 (0.0)	0 (0.0)	1 (4.2)
Gastrointestinal disorders	1 (16.7)	0 (0.0)	2 (25.0)	0 (0.0)	2 (8.3)
Diarrhea	1 (16.7)	0 (0.0)	2 (25.0)	0 (0.0)	2 (8.3)
General disorders and administration site conditions	0 (0.0)	0 (0.0)	0 (0.0)	1 (12.5)	1 (4.2)
Infusion site thrombosis	0 (0.0)	0 (0.0)	0 (0.0)	1 (12.5)	1 (4.2)
Infections and infestations	0 (0.0)	1 (12.5)	0 (0.0)	0 (0.0)	1 (4.2)
Upper respiratory tract infection	0 (0.0)	1 (12.5)	0 (0.0)	0 (0.0)	1 (4.2)
Injury, poisoning, and procedural complications	0 (0.0)	1 (12.5)	0 (0.0)	0 (0.0)	1 (4.2)
Arthropod bite	0 (0.0)	1 (12.5)	0 (0.0)	0 (0.0)	1 (4.2)
Investigations	4 (66.7)	5 (62.5)	4 (50.0)	6 (75.0)	15 (62.5)
Alanine aminotransferase increased	0 (0.0)	1 (12.5)	0 (0.0)	2 (25.0)	3 (12.5)
Amylase increased	0 (0.0)	1 (12.5)	1 (12.5)	1 (12.5)	3 (12.5)
Aspartate aminotransferase increased	0 (0.0)	0 (0.0)	0 (0.0)	2 (25.0)	2 (8.3)
Blood bilirubin increased	0 (0.0)	0 (0.0)	0 (0.0)	1 (12.5)	1 (4.2)
Blood calcium increased	0 (0.0)	0 (0.0)	1 (12.5)	0 (0.0)	1 (4.2)
Blood potassium increased	0 (0.0)	0 (0.0)	1 (12.5)	0 (0.0)	1 (4.2)
Blood pressure, diastolic, decreased	1 (16.7)	0 (0.0)	0 (0.0)	1 (12.5)	1 (4.2)
Blood pressure, diastolic, increased	0 (0.0)	0 (0.0)	0 (0.0)	1 (12.5)	1 (4.2)
Blood pressure, systolic, decreased	1 (16.7)	0 (0.0)	0 (0.0)	0 (0.0)	0 (0.0)
Blood pressure, systolic, increased	0 (0.0)	0 (0.0)	0 (0.0)	1 (12.5)	1 (4.2)
Hemoglobin decreased	1 (16.7)	2 (25.0)	1 (12.5)	1 (12.5)	4 (16.7)
Lipase increased	2 (33.3)	1 (12.5)	2 (25.0)	2 (25.0)	5 (20.8)
White blood cell count increased	1 (16.7)	0 (0.0)	0 (0.0)	0 (0.0)	0 (0.0)
Musculoskeletal and connective tissue disorders	0 (0.0)	0 (0.0)	1 (12.5)	0 (0.0)	1 (4.2)
Myalgia	0 (0.0)	0 (0.0)	1 (12.5)	0 (0.0)	1 (4.2)
Nervous system disorders	2 (33.3)	1 (12.5)	2 (25.0)	0 (0.0)	3 (12.5)
Dysgeusia	0 (0.0)	0 (0.0)	1 (12.5)	0 (0.0)	1 (4.2)
Headache	2 (33.3)	1 (12.5)	1 (12.5)	0 (0.0)	2 (8.3)
Psychiatric disorders	0 (0.0)	1 (12.5)	0 (0.0)	0 (0.0)	1 (4.2)
Anxiety	0 (0.0)	1 (12.5)	0 (0.0)	0 (0.0)	1 (4.2)
Respiratory, thoracic, and mediastinal disorders	0 (0.0)	0 (0.0)	1 (12.5)	1 (12.5)	2 (8.3)
Oropharyngeal pain	0 (0.0)	0 (0.0)	0 (0.0)	1 (12.5)	1 (4.2)
Throat irritation	0 (0.0)	0 (0.0)	1 (12.5)	0 (0.0)	1 (4.2)
Skin and subcutaneous tissue disorders	0 (0.0)	0 (0.0)	2 (25.0)	0 (0.0)	2 (8.3)
Dry skin	0 (0.0)	0 (0.0)	1 (12.5)	0 (0.0)	1 (4.2)
Erythema	0 (0.0)	0 (0.0)	1 (12.5)	0 (0.0)	1 (4.2)

a System organ class and preferred term are from the Medical Dictionary for Regulatory Activities (MedDRA), version 17.0. The number of subjects in each column could not be added because a subject may have had more than one adverse event. A subject experiencing multiple occurrences of the same adverse event was counted, at most, once per system organ class and preferred term for each treatment and once for “all active.” Q12h, every 12 h; TEAE, treatment-emergent adverse event.

**TABLE 3:** Summary of treatment-related adverse events by system organ class and preferred term for each treatment (safety population)^*a*^

System organ class/preferred term	No. (%) of subjects				
	Placebo (*n* = 6)	Cohort A (200 mg Q12h) (*n* = 8)	Cohort B (400 mg Q12h) (*n* = 8)	Cohort C (600 mg Q12h) (*n* = 8)	All active (*n* = 24)
Subjects with TEAEs	2 (33.3)	0 (0.0)	2 (25.0)	1 (12.5)	3 (12.5)
Gastrointestinal disorders	1 (16.7)	0 (0.0)	1 (12.5)	0 (0.0)	1 (4.2)
Diarrhea	1 (16.7)	0 (0.0)	1 (12.5)	0 (0.0)	1 (4.2)
Investigations	1 (16.7)	0 (0.0)	0 (0.0)	1 (12.5)	1 (4.2)
Alanine aminotransferase increased	0 (0.0)	0 (0.0)	0 (0.0)	1 (12.5)	1 (4.2)
Lipase increased	1 (16.7)	0 (0.0)	0 (0.0)	0 (0.0)	0 (0.0)
Nervous system disorders	0 (0.0)	0 (0.0)	1 (12.5)	0 (0.0)	1 (4.2)
Dysgeusia	0 (0.0)	0 (0.0)	1 (12.5)	0 (0.0)	1 (4.2)

a System organ class and preferred term are from the Medical Dictionary for Regulatory Activities (MedDRA), version 17.0. The number of subjects in each column could not be added because a subject may have had more than one adverse event. A subject experiencing multiple occurrences of the same adverse event was counted, at most, once per system organ class and preferred term for each treatment and once for “all active.” Related TEAEs are defined as TEAEs assessed as related to the study drug or those for which the relationship was unknown or missing.

### Microbiome analysis.

Of 266 stool samples processed for 16S rRNA gene sequencing, 248 samples resulted in adequate sequence data, yielding 2.1 million high-quality 16S rRNA gene V3-V4 region sequence reads that were used for taxonomic assignment.

**FIG 5:**
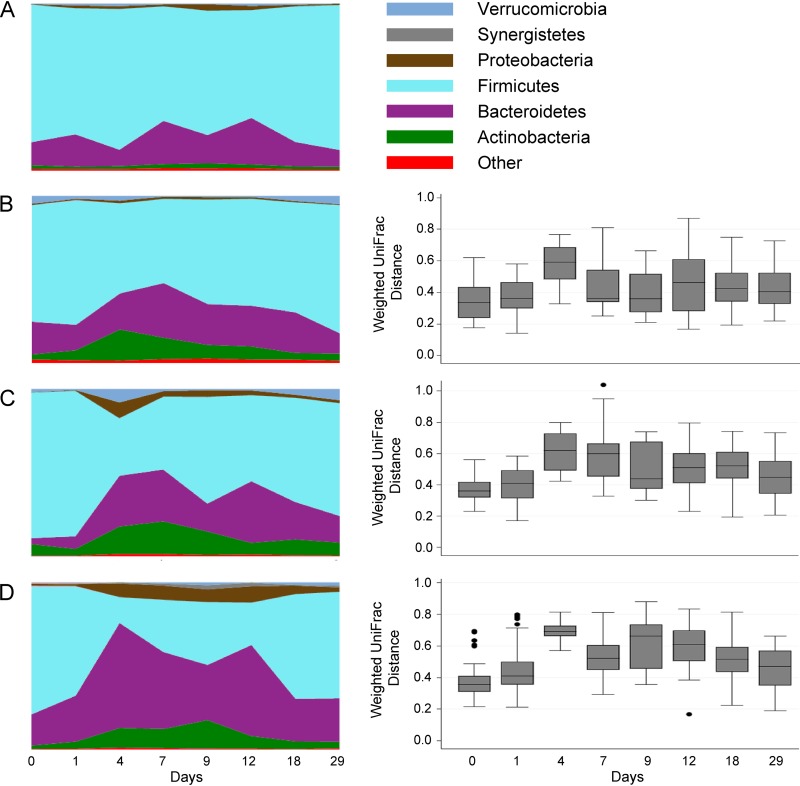
Effect of CRS3123 on the gut microbiota in healthy human subjects. Shown are taxon charts at the phylum level of placebo-treated subjects over the course of 29 days (A). Taxon charts at the phylum level and pairwise weighted UniFrac distances to placebo-treated subjects are shown for the 200-mg (B), 400-mg (C), and 600-mg (D) groups.

Analysis of community composition showed a significant difference between all treatment groups and the placebo arm at day 9 (point of highest difference) in a dose-dependent manner (median UniFrac distances, 0.361 [*P* = 0.006], 0.440 [*P* = 0.027], and 0.663 [*P* = 0.013] for 200 mg, 400 mg, 600 mg, respectively [Fig. 5]). However, all differences between the groups decreased by day 29 of the trial (median UniFrac distances, 0.404 [*P* = 0.006], 0.4480 [*P* = 0.177], and 0.470 [*P* = 0.077] for 200 mg, 400 mg, and 600 mg, respectively, 2 1/2 weeks after cessation of antibiotics). Increases in proteobacteria were minimal at 200 mg (maximum of 1.6%, compared to pretreatment value of 0.8%) and limited to <10% relative abundance at 600 mg.

**FIG 6:**
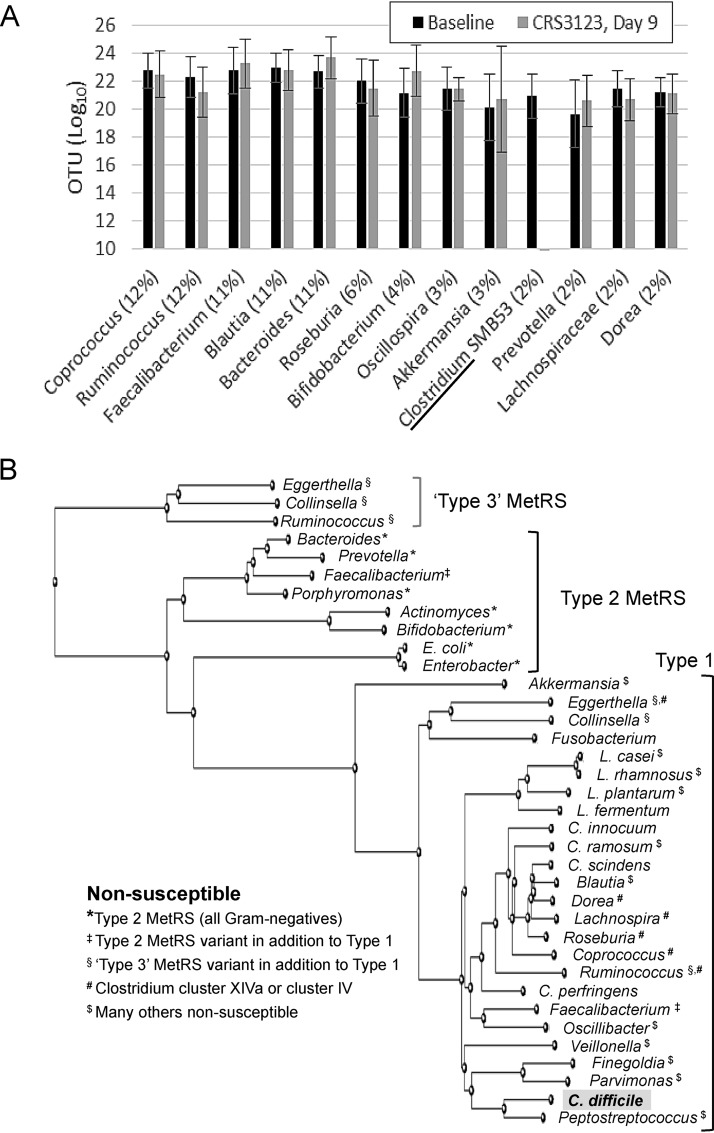
Effect of CRS3123 on prevalent genera (A) and phylogeny of MetRS in bacteria (B). These data are based on *in vitro* susceptibility results (31) and on microbiota analysis of stool samples collected during this phase 1 clinical trial.

The proportion of the major phyla in stool showed minimal changes over time in the 200-mg dosing group and was not different from that in the placebo group. In the 400-mg- and 600-mg-BID dosing groups, the relative abundance of *Firmicutes* decreased, while other phyla, such as *Bacteroidetes* and *Actinobacteria*, increased. Importantly, no phyla were lost during treatment with CRS3123 (Fig. 5) at any dose and the composition was back to baseline levels within 7 days of stopping the treatment. At the genus level, no significant changes were observed after 9 days of twice-daily dosing of CRS3123 in any of the highly abundant intestinal genera with the exception of the target genus *Clostridium* (Fig. 6A). CRS3123 did not impact commensal anaerobes, including *Bacteroides* and members of *Clostridium* clusters XIVa and IV (e.g., *Coprococcus*, *Dorea*, *Roseburia*, and *Ruminococcus*). The data obtained in microbiome analysis of healthy subjects during CRS3123 treatment are in good agreement with the expected spectrum of activity based on the MetRS phylogeny shown Fig. 6B.

## DISCUSSION

The study described in this report was preceded by a single-ascending-dose study of CRS3123, the first study in which a MetRS inhibitor was administered orally to humans, in single oral doses of 100 mg, 200 mg, 400 mg, 800 mg, and 1,200 mg (32). In this first-in-human study, CRS3123 was found to be safe and well tolerated, with no dose-limiting toxicities and no serious adverse events (SAEs) reported. The primary objective of the current study was to determine the safety and tolerability of escalating doses of CRS3123 following twice-daily oral administration to healthy adults over a period of 10 days. The secondary objectives were to determine the plasma, urine, and fecal concentrations and systemic exposure of CRS3123 after multiple oral doses, and the exploratory objective was to survey the effect of CRS3123 on the fecal microbiome.

CRS3123 administered in oral multiple ascending doses was generally safe and well tolerated, with no SAEs or severe TEAEs reported. No subjects were withdrawn from the study due to TEAEs. There were no trends in systemic, vital sign, or laboratory TEAEs. The majority of TEAEs reported in the study were of mild severity. There was no prolongation of the QTcF interval or any clinically significant changes in other ECG intervals and ECG morphology.

Absorption of CRS3123 was rapid, with median *T*_max_ occurring 2 to 3 h following a single dose and 1 to 2 h following multiple (twice-daily) dosing. The geometric mean *t*_1/2_ ranged from approximately 3 to 4 h following a single dose and from 5 to 6 h following multiple (twice-daily) dosing. There was essentially no accumulation with multiple (twice-daily) dosing, which is consistent with this relatively short *t*_1/2_ of CRS3123. In addition, the increase in CRS3123 exposure was less than proportional to the increase in dose across the dose range studied.

Following single- and multiple-dose administration of CRS3123, the majority of unchanged CRS3123 was retained in the GI tract and excreted in feces, while renal elimination of CRS3123 as free drug and glucuronide conjugates was minimal and accounted for less than 2% of the administered dose. The observation that high concentrations of CRS3123 are found in feces (well above 1,000 μg/g for all three doses tested) is important for the development of new agents for the treatment of CDI, since the lower GI tract is the site of the infection. Aside from efficacy potential, which is determined by the MIC_90_ value (1 μg/ml for CRS3123), equally important are the levels of the drug in relation to the mutant prevention concentration (MPC) value, which ranges from 16 to128 μg/ml, depending on the strain of C. difficile (29). As a class, tRNA synthetase inhibitors have been associated with either preexisting high-level resistance (as is the case of isoleucyl-tRNA synthetase inhibitor mupirocin resistance driven by a phylogenetically distinct enzyme encoded by the *mupA* gene) or rapid development of resistance during clinical development of epetraborole, a leucyl-tRNA synthetase inhibitor previously in development as a systemic agent for the treatment of urinary tract infections (33, 34). Fortunately, high-level resistance imparted by an entirely different MetRS isoform does not appear to exist in C. difficile, based on testing of more than 108 clinical isolates. This is worth noting, since some Gram-positive bacteria, notably Streptococcus pneumoniae and Bacillus anthracis, are known to express both type 1 and type 2 MetRS enzymes, leading to lack of susceptibility to all compounds in the same chemical series as CRS3123 (29). For suppression of emergence of strains with reduced susceptibility resulting from selection of spontaneous mutants, maintaining intraintestinal levels of drug well above the MPC throughout the dosing regimen is a key consideration, which, based on the measured CRS3123 concentrations in the feces reported in this study, is readily achievable.

Overall, the PK characteristics observed in human subjects suggest a promising profile for a CDI therapeutic; these properties include (i) limited systemic exposure and efficient clearance, both of which mitigate potential systemic safety risks, (ii) high concentrations achieved in the gut, well above the target MIC required for pharmacodynamic success, and (iii) high potential to suppress emergence of resistance due to the high ratio of gut concentration versus MPC.

Narrow antibacterial spectrum is of paramount importance for allowing rapid regeneration of healthy intestinal microbiota following an episode of CDI. CRS3123 has a low propensity for disruption of normal intestinal microbiota based on phylogenetic analyses of the MetRS gene as well as on *in vitro* studies (31). However, given that levels of CRS3123 present in the gut are much higher than concentrations tested in bacterial cultures (up to 32 μg/ml [31]), the impact of CRS3123 on the levels of the microbiome in healthy subjects was uncertain. We have shown here that CRS3123 indeed has minimal impact on the gut microbiome of healthy subjects.

The percentage of subjects with *Clostridium* species isolated at baseline but not isolated at subsequent visits was greater in the CRS3123 treatment groups than in the placebo group during the treatment phase of the study (day 1 through day 10). This provides preliminary evidence that CRS3123 has antimicrobial activity toward *Clostridium* species in healthy subjects. Individuals with chronic C. difficile infection often have very-low-diversity microbiomes that have increased abundances of facultative anaerobes such as proteobacteria (35); we did not see an increase in proteobacteria with the 200-mg and 400-mg doses of CRS3123 (Fig. 5A).

A narrow spectrum of activity is a prerequisite for optimal CDI therapy, allowing the compromised gut microbiota to recover and reestablish a healthy microbiome that provides protection from recurrence in the weeks following treatment. This is clearly a shortcoming of vancomycin, a broad-spectrum agent which is often used to treat CDI but results in recurrence in 25 to 30% of patients (25, 26). In fact, several recent drug candidates with a broad spectrum of activity, such as surotomycin, LFF-571, and cadazolid, failed to show reduced recurrence rates compared to that of vancomycin during clinical trials, halting their further development. In contrast, fidaxomicin and one agent in clinical development (ridinilazole) have a narrower spectrum and have demonstrated benefits with regard to reducing recurrences after treatment of CDI (36, 37). CRS3123 holds promise as the most narrow-spectrum agent against C. difficile yet, which is largely due to the phylogeny of the MetRS target. Gram-negative anaerobes in the gut microbiome, including *Bacteroides*, *Prevotella*, *Bifidobacterium*, *Porphyromonas*, and *Actinomyces* spp., harbor a MetRS type 2 enzyme resistant to CRS3123, which is consistent with the observed lack of *in vitro* or *in vivo* effect on these organisms. Among the Gram-positive anaerobes harboring the susceptible MetRS type 1 enzyme, several encode an additional MetRS type 2 (e.g., *Faecalibacterium* spp.) or yet another, phylogenetically distinct MetRS type 3 (e.g., *Eggerthella*, *Collinsella*, and *Ruminococcus*) which protects these beneficial species from CRS3123. Despite having only a type 1 MetRS, many other organisms of the normal gut microbiota were also nonsusceptible, including representatives of *Clostridium* clusters XIVa and IV (*Dorea*, *Lachnospira*, and *Roseburia*), which are important indicators of a healthy gut microbiome (38, 39), some *Lactobacillus* spp., and several other Gram-positives shown in Fig. 6. These data are based on *in vitro* susceptibility results for Clostridium ramosum, bifidobacteria, the Lactobacillus casei-L. rhamnosus-L. plantarum group, and Gram-negative anaerobes (31) and on microbiota analysis of stool samples collected during this phase 1 clinical trial. It is unknown whether resistance in these organisms is due to any of the subtle amino acid residue differences within their MetRS enzymes compared to the C. difficile MetRS and/or due to impaired uptake and/or efflux of the drug. It appears that the presence of MetRS type 1 is essential but not sufficient to confer susceptibility to CRS3123. In conclusion, CRS3123 demonstrated a substantially narrower spectrum than those of vancomycin and also to fidaxomicin, which are currently the only two FDA-approved drugs to treat CDI.

The results of this multiple-ascending-dose phase 1 study, together with an earlier single-ascending-dose phase 1 study, warrant the progression of testing of CRS3123 in patients with CDI.

## MATERIALS AND METHODS

### Study drug.

CRS3123 was provided by Crestone, Inc., in the form of dihydrochloride salt (CRS3123.2HCl) and formulated into 200-mg gelatin capsules by SRI International (Palo Alto, CA). Drug product stability specifications were met beyond the study period [stable for at least 60 months storage at room temperature monitored according to ICH Guidance for Industry Q1A(R2) Stability Testing of New Substances and Products].

### Study subjects and design.

This study was conducted in full accordance with the Declaration of Helsinki and good clinical practices (GCP) at Quintiles Phase One Services in Overland Park, KS. Subjects were randomized in a 4:1 fashion (8 active:2 placebo) in each cohort to receive 200 mg, 400 mg, or 600 mg of CRS3123 or matching placebo every 12 h for 10 days (https://clinicaltrials.gov/ct2/show/NCT02106338). Subjects fasted 1 h before and 2 h after administration of the study product with a minimum of 8 oz. (240 ml) of water. Water and noncaffeinated drinks (except grapefruit juice) during the study were not restricted. Informed consent was obtained before any study-related procedures and clinical evaluations were performed. All 30 randomized subjects completed the study; 24 subjects received CRS3123 and 6 subjects received placebo. There were no premature withdrawals or discontinuations.

After a screening period of up to 35 days, eligible subjects were admitted to the study center for baseline evaluations on day −1. On the morning of day 1, eligible subjects were randomized to receive either the study drug or placebo orally every 12 h through day 10. Subjects remained inpatient at the study center until discharge on day 12. During the inpatient period, subjects were monitored for safety and pharmacokinetics (PK). Subjects were followed for 2 additional outpatient visits at the study center, on days 18 ± 2 and 29 ± 2.

### Safety assessment and monitoring.

Subjects were monitored closely with serial physical examinations, blood evaluations, and 12-lead ECGs. Subjects underwent safety laboratory testing, including measurement of blood cell counts (white blood cell [WBC] with differential, hemoglobin, and platelets), serum chemistry (sodium, potassium, chloride, calcium, bicarbonate, glucose, creatinine, blood urea nitrogen [BUN], creatine kinase [CK], aspartate aminotransferase [AST], alanine aminotransferase [ALT], alkaline phosphatase [ALP], total bilirubin, protein, albumin, amylase, and lipase) and urinalysis (protein, blood, and glucose). Study halting criteria had been established and were reviewed by an independent safety monitor (ISM) and the Safety Monitoring Committee (SMC) prior to proceeding with dosing within a cohort or to the next cohort.

The safety population included all subjects who received at least one dose of the study drug. The safety and tolerability of CRS3123 were evaluated by the sequential review of reported adverse events (AEs), changes from baseline in physical examination findings, vital sign measurements, safety laboratory tests (hematology, clinical chemistry, and urinalysis), and key 12-lead ECG findings. All safety assessments were summarized with descriptive statistics or frequency counts.

The period of observation for AE reporting began on day 1 at the time of study drug administration and continued through the final visit. The following guidelines were used to quantify intensity: mild, events required minimal or no treatment and did not interfere with the subject’s daily activities); moderate, events resulted in a low level of inconvenience or were concerned with the therapeutic measures (moderate events may have caused some interference with functioning); severe, events interrupted a subject’s usual daily activity and required systemic drug therapy or other treatment (severe events are usually classified as incapacitating); and life-threatening, any adverse drug experience that, in the view of the investigator, placed the subject at immediate risk of death from the reaction as it occurred.

Relationship to study drug was assessed by the investigators based on evidence to suggest a causal relationship between the study drug and the AE. All AEs were coded to system organ class (SOC) and preferred term (PT) using the Medical Dictionary for Regulatory Activities (MedDRA), version 17.0 (https://www.meddra.org/). A treatment-emergent AE (TEAE) was defined as any AE that occurred during the study observation period (i.e., from day 1 at the time of study drug administration through the final visit). An AE with a partial onset date was classified as a TEAE if the reported partial onset date could not definitely be identified as before the date of study drug administration. Any AE with a missing onset date or missing time on day of study drug administration was classified as a TEAE.

To count the overall number of subjects who experienced each TEAE and calculation of an occurrence rate, a subject experiencing multiple occurrences of an AE was counted only once for that PT. Similarly, if a subject experienced multiple TEAEs within the same SOC, that subject was counted once to calculate an occurrence rate for the SOC. To count the number of subjects who experienced each TEAE by severity classification (mild, moderate, severe, and life-threatening) and calculate an occurrence rate, a subject experiencing multiple occurrences of an AE of the same severity was counted once in that particular severity classification for that PT. If a subject experienced multiple occurrences of an AE of differing severities, only the most severe occurrence within each PT was counted.

If any significant safety signals were encountered, the investigator was to notify the Division of Microbiology and Infectious Diseases (DMID), which would then determine the need for an *ad hoc* SMC review. For the purposes of this study, “*ad hoc*” simply means an unscheduled SMC review which could have been held at any time throughout the study per the DMID’s discretion. If deemed appropriate by halting rules and/or DMID’s discretion, enrollment was to be halted for an *ad hoc* SMC meeting. Decision to resume the study after halting was to be determined by DMID in consideration of the SMC recommendations. The dose for cohort B and study progression to cohorts B and C required a full (planned) SMC review of all safety data obtained through day 18. If the safety data review of the 200-mg dose had resulted in an unfavorable safety profile, the study design allowed for the reduction of the dose to 100 mg for cohort B and cohort C would not be dosed. However, no unfavorable safety profile was observed, and the dosing of cohort B at 400 mg and of cohort C at 600 mg was performed as planned.

The sample size for this study was not based on statistical considerations. Since safety testing of study drug was the primary objective of this study, consideration was given to the probability of detecting AE rates in 3 dosing groups of 10 subjects; each group size was logistically practical to provide sufficient information while minimizing the total number of subjects exposed to the study drug. There were no formal statistical hypotheses being tested in this study. With 8 active subjects in a cohort, an event rate of 24.5% or higher could be detected with 80% power.

### Study enrollment.

This study enrolled men and nonpregnant women 18 to 45 years of age, between June and December 2014. The subjects had to meet the following criteria to be included in the study: general good health, negative serum pregnancy test, negative alcohol and drug use screening, and body mass index (BMI) of <35 kg/m)^2^, agreement by subjects with reproductive potential to use an adequate method of contraception during the study and for 4 weeks after the initiation of study drug administration, and agreement to avoid strenuous exercise for at least 72 h prior to initial study drug administration and the scheduled follow-up visits on days 18 and 29.

Subjects with the following conditions were excluded from the study: hypertension, pulmonary disease, asthma, diabetes mellitus, autoimmune disorder, history of malignancy (except low-grade skin cancer, i.e., basal cell carcinoma, which was surgically cured), chronic renal, hepatic, or pulmonary disease or gastrointestinal tract condition that could interfere with the absorption of the study drug, history of known CDI, cardiac rhythm abnormalities, prolonged QT interval, or ovarian cysts. Also excluded were subjects with laboratory values outside the expanded ranges for the following tests: blood cell counts, serum chemistry, and urinalysis. In addition to these exclusion criteria, vulnerable populations were excluded from the study (https://clinicaltrials.gov/ct2/show/NCT02106338).

### Blinding.

The investigator and subjects remained blinded to individual subjects’ treatment assignments during the entire duration of the study. Blinding was not broken until all subjects had completed the final study visit, all PK analyses were completed, the database had been monitored and locked, and all results had been transferred for analysis.

### Analytical methodology.

PK method development, validation, and sample testing for plasma, feces, and urine were performed at KCAS, LLC (Shawnee, KS). Plasma, urine, and fecal concentrations of CRS3123 were determined by means of validated, sensitive, and specific high-performance liquid chromatography/tandem mass spectrometric (HPLC-MS/MS) assays. The lower limits of quantification of CRS3123 in plasma, feces, and urine were 10.0 ng/ml, 60.0 ng/g, and 100.0 ng/ml, respectively. The validated urine PK assay measured total free CSR3123. Since the majority of urinary CRS3123 was excreted as glucuronide conjugates (32), an enzymatic cleavage step using glucuronidase was added to the process to convert the glucuronide conjugates to free CRS3123 for the urine analysis.

A selective, accurate, and reproducible analytical method using LC-MS/MS for the quantitation of CRS3123 in human fecal homogenate was linear over a range of 10.0 to 2000 ng/ml (fecal homogenate) or 60.0 to 12,000 ng/g (fecal material). Since 11 fecal samples collected on day 10 exceeded the upper limit, this method was amended by validating it for an extended range of 50.0 to 3,000 μg/ml (fecal homogenate) or 300 to 18,000 μg/g (fecal material).

### Pharmacokinetics.

The PK population consisted of all subjects who received active study drug (CRS3123) and had at least one measured CRS3123 concentration at a scheduled PK time point after the start of study drug administration.

Pharmacokinetic parameters for plasma were derived using noncompartmental methods with Phoenix WinNonlin version 6.4 (Pharsight Corporation, Certara L.P., Princeton, NJ). PK parameters for urine and feces were derived using SAS version 9.4 (SAS Institute, Inc., Cary, NC). All statistical calculations and reporting were performed using SAS version 9.4. Graphics were prepared with SAS version 9.4 or SigmaPlot 12.5 (Systat Software, Inc., San Jose, CA).

**(i) Plasma PK.** Plasma concentrations of CRS3123 throughout the 12-h dosing interval on PK days (day 1 and day 10) were summarized using descriptive statistics for each treatment.

One-way analysis of variance (ANOVA) models were used to assess any differences in appropriate PK parameters between adjacent dose levels following a single dose (day 1) and multiple doses (day 10). An ANOVA model on the log-transformed PK parameters (AUC_0–12_ and *C*_max_ on day 1; AUC_0–tau_ and *C*_max_ on day 10) with fixed effect for treatment was performed separately for the single-dose analysis and multiple-dose analysis. From these models, the least-squares (LS) means together with 95% confidence intervals (Cis) for each dose and the LS means with corresponding 90% CIs for all adjacent dose pairs were calculated. Transformed back from the logarithmic scale, geometric LS means with corresponding 90% CIs were provided for each dose and adjacent dose ratios following both single and multiple dosing. PK accumulation was presented using descriptive statistics, and achievement of steady state was assessed graphically.

Dose proportionality of CRS3123 PK parameters following a single dose (AUC_0–12_ and *C*_max_ on day 1) and multiple doses (AUC_0–tau_ and *C*_max_ on day 10) over the administered dose range were quantified statistically using a power model ([AUC_0–12_ and *C*_max_ on day 1; AUC_0–tau_ and *C*_max_ on day 10] = α × dose^β^) for each PK parameter on log scale as the dependent variable and the logarithm of the dose as the independent variable. The model parameters (slope and intercept) were estimated using least-squares regression. Point estimates and corresponding 2-sided 90% CIs for the slope parameter (β) and the intercept parameter (α) were provided. A minimum of three values per dose level were required for a given parameter to estimate dose proportionality with the power model. Dose proportionality was concluded if the 90% CI for β fell within [ln(0.8)/ln(*R*), ln(1.25)/ln(*R*)], where *R* was the observed dose range for the study (600 mg/200 mg).

**(ii) Urine PK.** A listing of individual PK urine sample collection start and stop dates/times, volume of urine excreted over each planned collection interval, CRS3123 concentration in each urine collection, amount of CRS3123 excreted in each urine collection, and the fraction of dose excreted unchanged in urine was constructed for each urine collection by treatment. Urine samples for all 3 cohorts were stabilized by the bioanalytical laboratory upon receipt (for those samples which were not stabilized at the study center).

**(iii) Fecal PK.** Twenty-four-hour fecal collection was initiated for all subjects following dose 1 and dose 19 of study drug for measurement of CRS3123 concentrations in feces. A listing of individual PK fecal sample collection start and stop dates/times, fecal weight excreted for each planned collection, CRS3123 concentration in each fecal collection, and amount of CRS3123 excreted in each fecal collection was provided for each fecal collection for each treatment. The bioanalytical method was amended to allow for reanalysis of samples originally outside the linear range.

The following parameters were calculated from the fecal data: amount of unchanged drug excreted in feces (in nanograms) following doses 1 and 2 (*A*_e,feces_) or doses 19 and 20 (*A*_e,feces,ss_) calculated as fecal concentration × fecal weight (in the case of multiple fecal samples, *A*_e,feces_ and *A*_e,feces,ss_ were determined as the sum of the amount calculated in each fecal sample collected during the 24-h collection period), and fraction of dose excreted unchanged in feces (*f*_e,feces,ss_) over a 24-h interval after multiple dosing (expressed as a percentage), calculated as *A*_e,feces,ss_ divided by dose 19 plus dose 20.

Due to the 12-h dosing interval utilized in this study, *f*_e,feces,ss_ was calculated only after multiple dosing on day 10, when CRS3123 would be expected to be at or near steady state. These data were summarized using descriptive statistics. A subject listing of individual fecal PK parameters for each treatment was provided for day 1 and for day 10.

### Microbiome analysis.

Fecal microbiota profiles were measured for each sample as each operational taxonomic unit (OTU) species present and the relative abundance of that species, based on a region of 469 bp encompassing the V3 and V4 hypervariable regions of the 16S rRNA gene, following protocols developed at the Department of Microbiology and Immunology, University of Maryland School of Medicine Institute for Genome Sciences, Baltimore, MD (40–42).

**(i) Sample collection and storage and DNA extraction.** Fecal samples were collected at nine time points during the study for fecal microbiome analysis: twice before the first dose, on days 1, 4, 7, 9, and 12, and approximately 24 h prior to follow-up visits on day 18 and day 29. The subject was allowed to collect and bring the first fecal sample when admitted (collected within 24 h of admission and kept refrigerated until collected by staff). DNA was extracted from 150 mg of archived stool samples stored at –80°C for 16S rRNA gene amplification and sequencing. Cell lysis was initiated by adding 50 μl of lyzosyme (10 mg/ml), 6 μl of mutanolysin (25,000 U/ml; Sigma-Aldrich), and 3 μl of lysostaphin (4,000 U/ml in sodium acetate; Sigma-Aldrich) and 41 μl of TE50 buffer (10 mM Tris-HCl, 50 mM EDTA [pH 8.0]). Following a 1-h incubation at 37°C, 10 μl of proteinase K (20 mg/ml), 100 μl of 10% SDS, and 20 μl of RNase A (20 mg/ml) were added to the mixture and incubated for 1 h at 55°C. Microbial cells were lysed by mechanical disruption using a bead beater (FastPrep instrument; Qbiogene) set at 6.0 m/s for 30 s. DNA was purified from the lysate using the QIAsymphony robotics platform, consistently providing between 5 and 20 μg of high-quality and amplifiable whole genomic DNA. Following DNA quantitation via the Quant-iTTM PicoGreen double-stranded DNA (dsDNA) assay kit (Life Technologies), samples were diluted to a 5-ng/μl concentration.

### (ii) Amplification and sequencing of the 16S rRNA gene.

PCRs with dually barcoded 16S rRNA universal primers (319F/806R) were set up using a semiautomated platform with a ViaFlo 96 high-performance 96-channel pipettor and performed on Bio-Rad Tetrad 2 instruments with up to 576 dual index barcoding (40). Amplification was achieved with Phusion high-fidelity polymerase (New England BioLabs) and 50 ng of template DNA in a total reaction volume of 25 μl. The cycling parameters were 30 s of denaturation at 98°C, followed by 30 cycles of 15 s at 98°C (denaturing), 15 s at 58°C (annealing), and 15 s at 72°C (elongation), with a final extension at 72°C for 60 s. Controls included a no-template control for each barcode combination, extraction controls, and a DNA sample of known composition (positive control). The presence of amplicons was confirmed by gel electrophoresis on a 2% E-gel 96. PCR products were normalized using the SequalPrep normalization kit (Life Technologies, Carlsbad, CA) and pooled in equimolar amounts (25 ng) in a single tube. Amplification primers and reaction buffer were removed from the pooled amplicon mixtures using an AMPure kit (Agencourt). The purified amplicon mixtures (up to 576 per pool) were sequenced on an Illumina MiSeq instrument using the 300-bp paired-end protocol (MiSeq 300PE) at the Genomics Resource Center (GRC) at the Institute for Genome Sciences, University of Maryland School of Medicine.

**(iii) Bioinformatic analysis.** For sequence quality control, assembly, and taxonomic assignments, a custom bioinformatics pipeline (40) was applied that relies on QIIME software (43), UCHIME (44), and the RDP Naïve Bayesian classifier (45). Baseline for fecal microbiome assessments was defined as the mean of the predose assessments on day 1. Presence was defined as positive abundance. The presence of each OTU species and the shift in presence/absence occurrence from baseline were tabulated by treatment. Shifts were categorized as no change, appearance, and disappearance.

Additional 16S rRNA data analysis was performed at the Division of Biomedical Informatics and Personalized Medicine, University of Colorado. Joined and quality-filtered sequences sharing >97% sequence similarity were binned as OTUs. The OTUs were assigned taxonomical classification by SortMeRna (46) and sumaclust (47) to the Greengenes 13.8 database within QIIME 1.9 (43). Differences in community composition between placebo and treatment groups at each day were determined by weighted UniFrac (48) and permutational multivariate analysis of variance (PERMANOVA).

## Supplementary Material

Supplemental file 1
