# Supplementary material for: Multiple-Ascending-Dose Phase 1 Clinical Study of the Safety, Tolerability, and Pharmacokinetics of CRS3123, a Narrow-Spectrum Agent with Minimal Disruption of Normal Gut Microbiota
Source: Antimicrob Agents Chemother. 2019 Dec 20;64(1):e01395-19. doi: 10.1128/AAC.01395-19 (PMC7187627; doi:10.1128/AAC.01395-19)
Supplement: Supplemental file 1 [file AAC.01395-19-s0001.pdf]

Table S1. Study Demographics and Baseline Characteristics.

	Placebo (N = 6)	Cohort A (200 mg Q12h) (N = 8)	Cohort B (400 mg Q12h) (N = 8)	Cohort C (600 mg Q12h) (N = 8)	All Subjects (N = 30)
Sex (n [%])					
Female	2 (33.3%)	4 (50.0%)	2 (25.0%)	2 (25.0%)	10 (33.3%)
Male	4 (66.7%)	4 (50.0%)	6 (75.0%)	6 (75.0%)	20 (66.7%)
Race (n [%])[a]					
White	3 (50.0%)	4 (50.0%)	2 (25.0%)	4 (50.0%)	13 (43.3%)
Black or African American	2 (33.3%)	4 (50.0%)	6 (75.0%)	4 (50.0%)	16 (53.3%)
American Indian or Alaska Native	1 (16.7%)	0 (0.0%)	0 (0.0%)	0 (0.0%)	1 (3.3%)
Ethnicity (n [%])					
Hispanic or Latino	1 (16.7%)	1 (12.5%)	0 (0.0%)	0 (0.0%)	2 (6.7%)
Not Hispanic or Latino	5 (83.3%)	7 (87.5%)	8 (100.0%)	8 (100.0%)	28 (93.3%)
Age (years)					
Mean	35	28	28	29	30
SD	7	9	7	7	8
%CV	20.5	31	25.6	25.5	26.4
Minimum	25	19	22	21	19
Median	38	25	26	27	26
Maximum	43	44	44	41	44
Height (cm)					
Mean	171.4	169.8	174.5	169.8	171.4
SD	11.4	12.5	10.4	9.3	10.5
%CV	6.7	7.3	6	5.5	6.2
Minimum	154.2	153.5	160	155.5	153.5
Median	169.7	169.4	174.6	171.3	170
Maximum	188.6	188.9	186.7	181.4	188.9
Weight (kg)					
Mean	87	83.5	77.1	87.7	83.6
SD	7.3	24.2	9.5	13	15.2
%CV	8.4	28.9	12.3	14.9	18.2
Minimum	78.3	48	64.2	66	48
Median	86.5	85.4	76.8	89.4	83.9
Maximum	98	119.8	92.7	108.1	119.8
Body Mass Index (kg/m^2)					
Mean	29.79	28.47	25.48	30.28	28.42
SD	3.56	5.38	3.61	2.45	4.18
%CV	11.9	18.9	14.2	8.1	14.7
Minimum	25.7	19.84	18.46	26.44	18.46
Median	29.76	30.33	26.22	31.01	29.69
Maximum	34.19	33.57	31.21	33.66	34.19

[a] No subjects of Asian, Native Hawaiian or Other Pacific Islander ethnicity were enrolled in the study.

Table S2. Plasma CRS3123 Pharmacokinetic Parameters by Treatment – Day 1.

	$AUC_{(0-12)}$ (ng·h/mL)	C_{max} (ng/mL)	t_{max} (h)	$t_{1/2}$ (h)
**Cohort A (200 mg Q12h)**				
n	8	8	8	7
Geo mean	1550	352	ND	3.01
Geo %CV	64.1	59.4	ND	10.2
Minimum	921	183	0.50	2.73
Median	1230	342	2.00	2.96
Maximum	4880	896	4.00	3.71
**Cohort B (400 mg Q12h)**				
n	8	8	8	4
Geo mean	2340	507	ND	3.53
Geo %CV	57.6	42.5	ND	23.7
Minimum	1050	275	2.00	2.87
Median	2510	504	3.00	3.61
Maximum	5430	1040	4.00	4.33
**Cohort C (600 mg Q12h)**				
n	8	8	8	6
Geo mean	3560	654	ND	3.04
Geo %CV	64.1	57.9	ND	16.5
Minimum	1490	310	1.00	2.61
Median	3630	642	2.00	2.85
Maximum	8420	1210	4.02	4.02

CV%: Coefficient of variation; Geo: Geometric; ND: Not determined; Q12h: Every 12 hours; SD: Standard deviation.

Table S3. Plasma CRS3123 Pharmacokinetic Parameters by Treatment – Day 10.

	$AUC_{(0\text{-}tau)}$ (ng·h/mL)	$C_{max,ss}$ (ng/mL)	$C_{min,ss}$ (ng/mL)	$t_{max,ss}$ (h)	$t_{1/2,ss}$ (h)	$CL/F_{,ss}$ (L/h)	$V_z/F_{,ss}$ (L)	RAUC	RC_{max}
**Cohort A (200 mg Q12h)**									
n	8	8	8	8	7	8	8	8	8
Geo mean	2500	470	85.2	ND	4.71	80.2	591	1.61	1.34
Geo %CV	41.3	32.3	56.9	ND	18.4	41.3	29.0	46.1	49.1
Minimum	1540	320	44.2	1.00	4.14	44.7	354	0.916	0.781
Median	2470	492	76.2	1.50	4.59	82.0	578	1.54	1.11
Maximum	4470	837	202	2.00	6.99	130	888	3.74	2.70
**Cohort B (400 mg Q12h)**									
n	8	8	8	8	8	8	8	8	8
Geo mean	3200	615	112	ND	5.19	125	937	1.37	1.22
Geo %CV	29.4	32.4	41.1	ND	28.5	29.5	31	37.4	20.9
Minimum	2000	407	53.8	1.05	3.4	77.2	606	0.954	0.830
Median	3310	603	122	2.00	5.01	121	917	1.21	1.19
Maximum	5180	1100	179	2.00	7.71	200	1600	2.41	1.52
**Cohort C (600 mg Q12h)**									
n	8	8	8	8	7	8	8	8	8
Geo mean	4030	731	160	ND	6.32	149	1400	1.13	1.12
Geo %CV	55.6	47.9	53.7	ND	11	55.6	66.5	38.1	39.9
Minimum	1750	319	73.2	1.00	5.36	69.3	600	0.663	0.626
Median	3740	674	161	1.00	6.1	162	1410	1.15	1.05
Maximum	8660	1390	313	2.00	7.3	343	3570	1.68	2.09

CV%: Coefficient of variation; Geo: Geometric; ND: Not determined; Q12h: Every 12 hours; SD: Standard deviation.

Table S4. Descriptive Statistics for CRS3123 Fecal Pharmacokinetic Parameters for Each Treatment – Day 1 and Day 10

	Day 1	Day 10	
	$A_{e,feces}$ (ng)	$A_{e,feces,ss}$ (ng)	$f_{e,feces,ss}$ (%)
**Cohort A (200 mg Q12h)**			
Evaluable n	6	8	8
Mean	1.90×10^{7}	1.18×10^{8}	29.6
SD	3.25×10^{7}	8.81×10^{7}	22.0
%CV	171	74.5	74.5
Minimum	5.19×10^{3}	7.71×10^{6}	1.90
Median	4.97×10^{5}	9.97×10^{7}	24.9
Maximum	7.96×10^{7}	2.75×10^{8}	68.6
**Cohort B (400 mg Q12h)**			
Evaluable n	4	8	8
Mean	8.38×10^{5}	4.03×10^{8}	50.4
SD	1.67×10^{6}	2.21×10^{8}	27.6
%CV	199	54.7	54.7
Minimum	0.00	2.84×10^{7}	3.5
Median	5.15×10^{3}	4.16×10^{8}	52.0
Maximum	3.34×10^{6}	7.01×10^{8}	87.6
**Cohort C (600 mg Q12h)**			
Evaluable n	7	8	8
Mean	3.74×10^{7}	6.33×10^{8}	52.7
SD	6.25×10^{7}	4.61×10^{8}	38.4
%CV	167	72.8	72.8
Minimum	0.00	1.31×10^{8}	11.0
Median	5.97×10^{4}	5.17×10^{8}	43.1
Maximum	1.66×10^{8}	1.51×10^{9}	125.8

$A_{e,feces}$: Amount of unchanged drug excreted in feces (ng) following Doses 1 and 2 calculated as [fecal concentration × fecal weight]. In the case of multiple fecal samples, $A_{e,feces}$ was determined as the sum of the amount calculated in each fecal sample collected during the 24-hour collection period.

$A_{e,feces,ss}$: Amount of unchanged drug excreted in feces (ng) following Doses 19 and 20, calculated as [fecal concentration x fecal weight]. In the case of multiple fecal samples, $A_{e,feces,ss}$ was determined as the sum of the amount calculated in each fecal sample collected during the 24-hour collection period.

$f_{e,feces,ss}$: Fraction of dose excreted unchanged in feces over a 24-hour interval after multiple dosing (%), calculated as $A_{e,feces,ss}$ divided by Dose 19 + Dose 20.

Geometric mean and Geometric %CV values were not calculated due to high inter-subject variability, especially on day 1, with zero values observed in some subjects.

Table S5. Descriptive Statistics for CRS3123 Urine Pharmacokinetic Parameters for Each Treatment – Day 1 and Day 10

	Day 1		Day 10	
	$A_{e,\ (0\text{-}12),\ urine}$ (ng)	$f_{e,\ (0\text{-}12),\ urine}$ (%)	$A_{e,\ (0\text{-}12),\ ss,\ urine}$ (ng)	$f_{e(0\text{-}12),ss,\ urine}$ (%)
**Cohort A (200 mg Q12h)**				
n	8	8	7	7
Mean	2.27×10^6	1.13	3.80×10^6	1.90
SD	5.56×10^5	0.278	8.76×10^6	0.439
%CV	24.5	24.5	23.1	23.1
Minimum	1.55×10^6	0.778	2.64×10^6	1.32
Median	2.30×10^6	1.15	3.38×10^6	1.69
Maximum	33.06×10^6	1.53	4.92×10^6	2.46
Geometric mean	2.20×10^6	1.10	3.71×10^6	1.85
Geometric %CV	25.7	25.6	23.7	23.7
**Cohort B (400 mg Q12h)**				
n	8	8	8	8
Mean	4.29×10^6	1.07	5.36×10^6	1.34
SD	1.83×10^6	0.459	9.44×10^5	0.237
%CV	42.7	42.8	17.6	17.7
Minimum	2.38×10^6	0.596	4.11×10^6	1.03
Median	4.17×10^6	1.05	5.26×10^6	1.32
Maximum	8.22×10^6	2.06	7.26×10^6	1.82
Geometric mean	4.00×10^6	1.00	5.30×10^6	1.32
Geometric %CV	40.7	40.7	17.1	17.1
**Cohort C (600 mg Q12h)**				
n	8	8	8	8
Mean	$5.39 \times ^{106}$	0.899	6.96×10^6	1.16
SD	2.36×10^6	0.393	2.14×10^6	0.358
%CV	43.8	43.8	30.7	30.8
Minimum	2.86×10^6	0.476	3.32×10^6	0.553
Median	4.25×10^6	0.708	6.63×10^6	1.11
Maximum	8.90×10^6	1.48	9.88×10^6	1.65
Geometric mean	4.98×10^6	0.829	6.63×10^6	1.11
Geometric %CV	44.4	44.4	35.8	35.9

For Subject 1002 (Cohort A), urine was not collected on Day 10 for the 0 – 4 hour interval.

$A_{e,urine(0\text{-}12)}$: Cumulative amount of drug (free and glucuronide conjugates) excreted in urine from zero (predose) to 12 hours (ng), calculated as the summation of the amounts excreted in the scheduled collection intervals through the 12 hour dosing interval following Dose 1.

$A_{e,urine(0\text{-}12),ss}$: Cumulative amount of drug (free and glucuronide conjugates) excreted in urine from zero (predose) to 12 hours after multiple dosing (ng), calculated as the summation of the amounts excreted in the scheduled collection intervals through the 12 hour dosing interval following Dose 19.

$f_{e,urine(0\text{-}12)}$: Fraction of dose excreted in urine from zero (predose) to 12 hours (%), calculated as Ae,urine(0-12) divided by Dose 1.

$f_{e,urine(0\text{-}12),ss}$: Fraction of dose excreted in urine from zero (predose) to time tau after multiple dosing (%), calculated as $A_{e,urine(0\text{-}12),ss}$ divided by Dose 19.
